# ABCG1 rs57137919G>A Polymorphism Is Functionally Associated with Varying Gene Expression and Apoptosis of Macrophages

**DOI:** 10.1371/journal.pone.0097044

**Published:** 2014-06-27

**Authors:** Fang Liu, Wei Wang, Yan Xu, Yu Wang, Lian-Feng Chen, Quan Fang, Xiao-Wei Yan

**Affiliations:** Department of Cardiology, Peking Union Medical College Hospital, Chinese Academy of Medical Sciences and Peking Union Medical College, Beijing, China; Ohio State University Medical Center, United States of America

## Abstract

ATP-binding cassette transporter G1 (ABCG1) is a transmembrane cholesterol transporter involved in macrophage sterol homeostasis, reverse cholesterol transport (RCT), and atherosclerosis. The role of ABCG1 in atherosclerosis remains controversial, especially in animal models. Our previous study showed that single nucleotide polymorphism rs57137919 (-367G>A) in the ABCG1 promoter region was associated with reduced risk for atherosclerotic coronary artery disease (CAD). This study was designed to provide functional evidence for the role of rs57137919G>A in atherosclerosis in humans. We combined *in* *vitro* and *ex* *vivo* studies using cell lines and human monocyte-derived macrophages to investigate the functional consequences of the promoter polymorphism by observing the effects of the rs57137919A allele on promoter activity, transcription factor binding, gene expression, cholesterol efflux, and apoptosis levels. The results showed that the rs57137919A allele was significantly associated with decreased *ABCG1* gene expression possibly due to the impaired ability of protein-DNA binding. ABCG1-mediated cholesterol efflux decreased by 23% with rs57137919 A/A versus the G/G genotype. Cholesterol-loaded macrophage apoptosis was induced 2-fold with the A/A genotype compared with the G/G genotype. Proapoptotic genes *Bok* and *Bid* mRNA levels were significantly increased in macrophages from the A/A genotype compared with those from the G/G genotype. These findings demonstrated that the ABCG1 promoter rs57137919G>A variant had an allele-specific effect on ABCG1 expression and was associated with an increased apoptosis in cholesterol-loaded macrophages, providing functional evidence to explain the reduced risk for atherosclerosis in subjects with the ABCG1 promoter rs57137919A allele as reported in our previous study.

## Introduction

Atherosclerosis is characterized by the accumulation of lipids in the subendothelium of large and medium-sized arteries, which results in plaque formation and arterial narrowing [1]–[3], while deposition of excessive lipid-loaded macrophage foam cells in the arterial intima is a pathological hallmark of early fatty streak lesions. Several ATP-binding cassette (ABC) transporters, including ABCG1, have been involved in macrophage sterol homeostasis, reverse cholesterol transport, and atherosclerosis [4], [5]. Kennedy et al. first described that *Abcg1* ^−/−^ mice accumulated excessive cholesterol in macrophages of multiple tissues, especially in the lung [6]. Early studies indicated that overexpression of ABCG1 in primary cells or cell lines lead to increased cholesterol efflux to high density lipoprotein (HDL) or low density lipoprotein (LDL) [6]–[9]. These results strongly suggested that ABCG1 might play a vital role in sterol efflux from cholesterol-loaded macrophages to HDL, which is the first critical step of RCT. Therefore, it was presumed that *ABCG1* knockout in macrophages would result in increased foam cells and atherosclerosis. However, the results of *ABCG1* knockout studies in animal models are not consistent. Ranalletta et al. [10] and Baldan et al. [11] simultaneously reported that hyperlipidemic *Ldlr^−/−^* mice transplanted with *Abcg1* ^−/−^ bone marrow exhibited a significant reduction in atherosclerotic lesions compared with *Ldlr^−/−^* mice receiving *Abcg1* ^+/+^ bone marrow. On the other hand, Out et al. reported a moderate increase in atherosclerotic lesions which may result from reduced efflux of cholesterol from the macrophages in *ABCG1* deleted mice [12]. Therefore, the role of ABCG1 in atherosclerosis remains controversial, especially in animal models.

Genetic association studies in human have helped identify the role of disease candidate genes [13]. We have previously performed a case-control association analysis with hospital-based atherosclerotic coronary artery disease (CAD) samples to investigate the association of *ABCG1* polymorphisms with the risk of atherosclerotic CAD by a candidate gene approach [14]. In that study, four nucleotide variants of the *ABCG1* gene locus in the Chinese Han population were identified in 1,021 CAD patients and 1,013 control subjects. Our study revealed that, among the four single nucleotide polymorphisms (SNPs), rs57137919G>A, which is located in the promoter region, was associated with a decreased susceptibility to CAD and was even more evident with respect to the prevalence of multi-vessel CAD. In that preliminary study, we also found that macrophage ABCG1 protein expression was significantly lower in subjects carrying the A allele of rs57137919. Our results strongly suggested that ABCG1 expressed in human macrophages might be potentially atherogenic. In their commentary of our article, LeGoff et al. pointed out that the study highlighted the complex role of *ABCG1* in biological processes leading to atherosclerosis and that further investigations needed to be conducted to understand the function of the *ABCG1* gene in atherosclerosis development in humans [15].

Herein, we present functional evidence demonstrating that the promoter SNP of the intracellular lipid transporter gene *ABCG1* might lead to a discrepancy, not only in the level of gene expression, but also in cholesterol efflux and apoptosis in macrophages. Through the combination of our present observations and previous findings from the case-control association study, we infer that rs57137919-associated phenotypic differences could contribute to individual susceptibility to atherosclerosis.

## Materials and Methods

### Study population

A total of 200 healthy volunteers were recruited from the Medical Examination Center of Peking Union Medical College Hospital (PUMCH). Subjects (men or women) were all between 18 and 55 years old and their body mass index ranged from 18 to 25 kg/m^2^. Study protocols were reviewed and approved by the Ethics Committee of PUMCH. Each participant gave written informed consent. Peripheral blood samples were obtained from study subjects after fasting for at least 12 hours. The ethylenediaminetetraacetic acid anticoagulated blood samples for genotype analysis were immediately centrifuged and stored at –80°C until batch analysis. Heparinized blood was sampled and human peripheral blood mononuclear cells (PBMCs) were isolated and cultured within 6 hours.

### *ABCG1* genotyping

Genomic DNA samples were extracted from whole blood using Blood Genomic DNA Kit (EasyPure, TansGen Biotech, China) in accordance to the manufacturer’s instructions. Polymerase chain reaction (PCR) was used to generate 684 bp fragments from human ABCG1 promoter around the rs57137919 site using the following primers: 5′-GAGTGTGCCCTGGTGTTATTC-3′ and 5′-GTGCTCTTGTTTTGCTCCTGC-3′. The PCR products were purified and subsequently sequenced using 3730XL DNA Analyzer (Applied Biosystems). In this study, 200 subjects were collected and genotyped as A/A, G/A, and G/G groups in accordance with ABCG1 promoter rs57137919G>A polymorphism by analysis using Chromas software.

### Cells and cell culture

Human embryonic kidney (HEK) 293T, HepG2, CHO-K1, and Human acute monocytic leukemia (THP-1) cell lines were obtained from the American Type Culture Collection (ATCC, Manassas, VA, USA). All cells were incubated at 37°C with 5% CO_2_ and 95% humidity. HEK 293, HepG2, and CHO-K1 cells were cultured in DMEM with 10% fetal bovine serum (FBS, Gibco, Invitrogen). THP-1 cells were cultured in RPMI-1640 with 10% FBS and pretreated with 100 ng/mL phorbol-12-myristate-13-acetate (PMA, Sigma-Aldrich, St Louis, MO, USA) for 24 hours for monocytic differentiation before transfection. PBMCs were isolated by density gradient centrifugation after layering on Ficoll-Paque Plus (GE healthcare, NJ, USA) and 2–5×10^6 ^PBMCs were plated into 12-well plastic plates (Costar) in RPMI-1640 medium with 10% autologous serum and 2 mmol/L glutamine. After 24 hours, non-adherent cells were removed. Adherent monocytes were differentiated into macrophages by culture in RPMI-1640 supplemented with 10% autologous serum, 2 mmol/L glutamine, and 20 ng/mL recombinant human macrophage colony-stimulating factor (M-CSF, Peprotech, Rocky Hill, NJ, USA) for 7 days.

### Construction of luciferase reporter gene and activity assay

**Figure 1:**
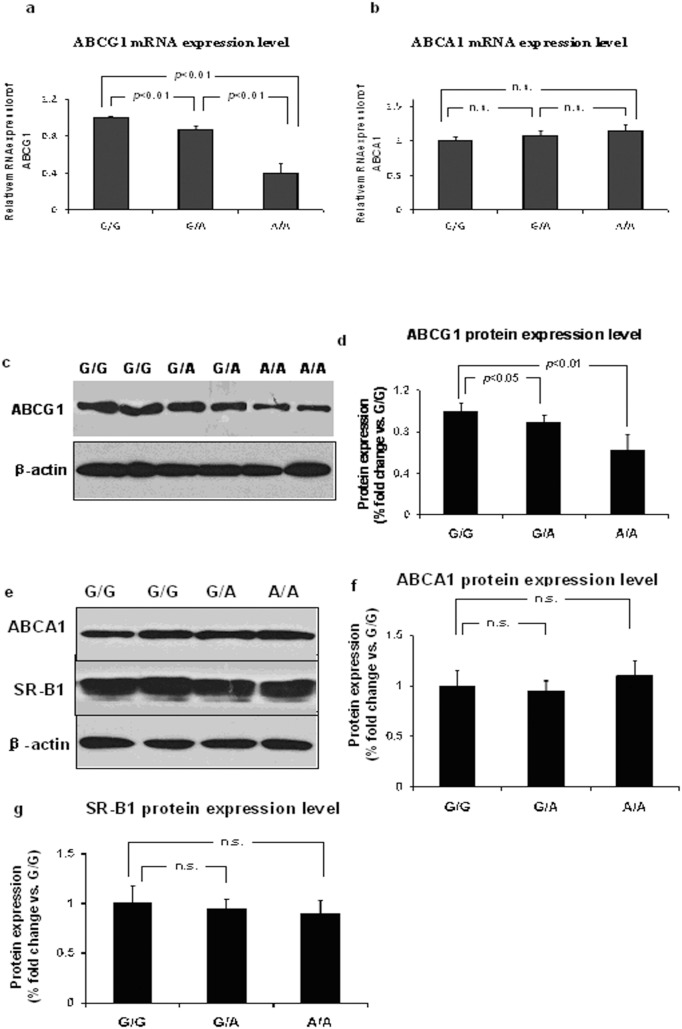
Luciferase reporter analysis of rs57137919G or A constructs of *ABCG1* promoter. (a) Schematic diagram for constructing wild-type or mutant promoter at the *ABCG1* rs57137919G>A polymorphism site. Two constructs were subcloned into pGL3-basic plasmid vectors with firefly luciferase reporter gene. pG, -367G (open bar); pA, -367A (grey bar). Luciferase activity assays for two ABCG1 promoter constructs in HEK293T cells (b), THP-1 cells (c), and HepG2 cells (d) are shown. Luciferase activities were measured with the dual-luciferase reporter assay system and normalized to Renilla luciferase activity 24 hours after transfection in the presence or absence of TO901317. The luciferase activity was a corrected relative value. Data are shown as mean ± SD of four independent experiments in triplicate. *P*<0.01 vs. pG in HEK293 cells, THP-1 cells, and HepG2 cells under both basal state and LXR agonist stimulation.

Human *ABCG1-*367A and *ABCG1-*367G promoter constructs were generated as previously described [14] (Figure 1a). A PCR product with wild-type or mutation was then cloned into pGL3-basic reporter vectors with firefly luciferase gene (Promega, Madison, WI, USA); the two constructs were verified by sequencing. Luciferase activity assay for the two *ABCG1* promoter constructs was conducted in cultured HEK293T, HepG2, and THP-1 cells. Under basal state or intervention of 2 µM TO901317 for 24 hours, cells were transfected in each well of a 48-well plate with 1 µg of each plasmid construct and 5 ng of Renilla luciferase as phRL-TK control plasmid using Fugene HD (Promega). The pGL3-basic empty vector was used as a negative control and the pGL3-control vector (Promega) was used as a positive control for the luciferase assay. Luciferase activities were measured with Promega dual-luciferase reporter assay system 24 hours after transfection. Firefly luciferase activities were normalized to Renilla luciferase activities.

### RNA isolation and real-time PCR

Total RNA was obtained from macrophages of subjects with the G/G, G/A, and A/A genotypes of rs57137919 using the TRIzol regent (Invitrogen). RNA was extracted following the manufacturer’s instructions. The purity of the RNA preparation was checked by measuring the absorbance ratio at 260/280 nm and 1 µg of RNA was used for reverse transcription (RT) according to the manufacturer’s recommendations (Superscript II Reverse Transcriptase and Random Hexamers, Invitrogen). Quantitative RT-PCR amplifications (ABI PRISM 7500, Applied Biosystems) were performed in triplicate at 40 cycles of 95°C for 5 s and 60°C for 34 s using SYBR Premix Ex Taq II (Takara Biotech, Japan). Primers used for RT-PCR are shown in Table 1. Data analysis was performed using 2^−△△Ct^ base fold-change calculations and normalized for GADPH expression.

**Table 1:** Primers used for real-time PCR.

Gene	Genbank accession no.	Sequences	Product length (bp)
hABCA1	NM_005502	Forward 5′ TACAGCCAGAAAGACACCAG 3′	164
		Reverse 5′ CACAGTAGACTTTGGGAGAG 3′	
hABCG1	NM_004915	Forward 5′ GAGGGATTTGGGTCTGAAC 3′	259
		Reverse 5′ GCAGCCTTCCATGGACGA 3′	
hBok	NM_032515	Forward 5′ CGCCTTTGACCGCTCGCCCACAGA 3′	167
		Reverse 5′ GCAGCAGCACCGCGCACACCTCAG 3′	
hBid	NM_001196	Forward 5′ CGCCGTCCTTGCTCCGTGAT 3′	189
		Reverse 5′ ACATGCCAGGGCTCCGTCTACA 3′	
hGAPDH	NM_002046	Forward 5′ CTCTGCTCCTCCTGTTCGAC 3′	111
		Reverse 5′ ACGACCAAATCCGTTGACTC 3′	

### Western blot analysis

Whole cell proteins were extracted with RIPA buffer (Applygen Technologies Inc., China). The protein concentrations were determined by bicinchoninic acid (BCA) protein assay (Pierce Perbio Science). Equal amounts of cell protein were separated using 10% SDS-PAGE gels and the proteins were transferred onto PVDF membranes. Membranes were blotted using a rabbit anti-ABCG1 monoclonal antibody (2,000× dilution; Abcam) followed by anti-mouse or anti-rabbit HRP-conjugated secondary antibodies (10,000× dilution, Santa Cruz, CA, USA) and visualized using an enhanced chemiluminescence technique (ECL, Amersham Biosciences, California, USA). The intensity of the bands was analyzed using the Image J scanning software.

### Cholesterol efflux assay

The cholesterol efflux assay was performed using 22-[N-nitrobenz -2-oxa-1,3-diazol-4-yl-amino]-23,24-bisnor-5-cholen-3b-ol (NBD-cholesterol; Invitrogen, USA) as previously described [16], [17]. Briefly, monocyte-derived macrophages were isolated and cultured for 7 days before loading with ox-LDL 50 µg/mL (Peking Union-Biology Co. Ltd., Beijing, China) for 24 hours. Cells were then labelled with NBD-cholesterol (2 µM) in culture medium for 4 hours. The cells were washed three times and incubated with 50 µg/mL HDL (Sigma-Aldrich) in RPMI-1640 medium containing 0.2% bovine serum albumin (BSA, Sigma-Aldrich) to induce cholesterol efflux. After 4 hours, aliquots of the medium were transferred into 96-well black polystyrene assay plates (Costar, Corning, Inc.). Cells were lysed with 0.1% TritonX-100 for 30 min after washing with cold PBS. Fluorescence intensity from the medium and the cell lysate were determined at excitation and emission wavelengths of 469 and 537 nm, respectively, using Biotek Synergy H1 (USA). The percentage of cholesterol efflux was calculated by dividing the fluorescence value of the medium by the sum of the fluorescence value in the cell lysate and medium. To calculate the net ABCG1-mediated cholesterol efflux, the cholesterol efflux of untreated cells was subtracted from the efflux of the cells treated with HDL.

### Electrophoretic mobility shift assay (EMSA)

The following probe sequences were designed: Biotinylated Probe G: 5′-Bio-CA GCCCC CGCGA**G**TTCG GGACCCG-3′; unlabelled G probe: 5′-CAGCCCCCGCGA **G**TTCG GGACCCG-3′; biotin-labelled A probe: 5′-Bio-CAGCCCC CGCGA**A**TTCG GGACCCG-3′; unlabelled A probe: 5′-CAGCCCC CGCGA**A**TTCG GGACCCG-3′ (Invitrogen). THP-1 and monocyte-derived macrophage nuclear protein were extracted using a nucleoprotein extraction kit (Viagene Biotech, USA). Protein concentration was determined by the BCA method. Biotin-labelled G or A probes (0.3 pmol) and nuclear protein extracts (5–15 µg) were added to 15 µL of total binding reaction system containing 10 mmol/L Tris-HCL (pH 7.5), 1 mmol/L MgCl_2_, 50 mmol/L NaCl, 0.5 mmol/L EDTA, 0.5 mmol/L dithiothreitol, 4% (v/v) glycerol, and 0.05 mg/mL Poly (dI:dC) (Promega). To test the specificity of DNA-protein interaction, nuclear proteins were incubated for 15 min with a 20- or 100-fold molar excess of unlabelled cold DNA probe followed by addition of the labelled DNA probes, thus performing self- and cross-competition assays. After 20 min of incubation at room temperature, the mixture was added onto a 6.5% non-denaturating polyacrylamide gel containing 2.5% (v/v) glycerol in 0.5× TBE buffer and electrophoresed in 0.25× TBE buffer at 180 V for 60 min at 4°C to separate DNA-protein complexes. Complexes were transferred onto a Biodyne nylon membrane (Pierce) by electroblotting in 0.5× TBE buffer at 400 mA for 40 min. Following ultraviolet cross-linking for 10 min, the ladder of bands was visualized by using a LightShift chemiluminescence EMSA kit (Pierce, Rockford, IL, USA) following the manufacturer’s protocol.

### Apoptosis assay

PBMC-derived macrophages were plated in 12-well culture plates in triplicate and incubated in RPMI-1640 medium supplemented with ox-LDL (50 µg/mL) and 0.2% BSA for 24 hours. Cells were then detached with a rubber policeman and rinsed twice in cold PBS. Apoptosis was determined using PE-Annexin V and 7-AAD double staining assay (BD Biosciences, Palo Alto, CA, USA) according to the manufacturer’s instructions. Cells were counted immediately with flow cytometry using Accuri C6 flow cytometry (BD Biosciences). Data were analyzed by CFlow Plus software (BD Biosciences) and apoptosis was presented as the percentage of 7-AAD-negative/Annexin V-positive cells. The level of proapoptotic genes *Bid* and *Bok* mRNA in macrophages after incubation with ox-LDL was analyzed using quantitative real time PCR as described under the “RNA isolation and real-time PCR” section.

### Statistical analysis

Statistical analysis was carried out by using GraphPad Prism Software Version 3.0 (San Diego, CA, USA). Data are presented as mean ± SD. Comparisons between two groups were analyzed using unpaired, two-tailed student *t*-test. Multiple groups were compared by one-way analysis of variance (ANOVA) with a Bonferroni post hoc test. A value of *P*<0.05 was considered statistically significant.

## Results

### *ABCG1*-367G>A haplotype analysis

In 200 subjects, the G/G, G/A, and A/A genotypes were observed in 116, 75, and 9 cases, respectively. In this study, 62 donors with different genotypes were randomly selected from the 200 subjects to collect blood samples and obtain PBMCs. Age,gender and the lipid level profiles, including total cholesterol (TC), triglycerides (TG), HDL cholesterol, and LDL cholesterol, showed no statistical difference among the three groups. The general characteristics of the study participants are summarized in Table 2.

**Table 2:** General characteristics of subjects in different *ABCG1* genotype groups.

Genotype: -367G>A	G/G	G./A	A/A
No. of subjects	28	25	9
Sex, female/male	17/11	18/7	4/5
Age, years	29±4	31±5	34±6
BMI (kg/m^2^)	23.6±2.3,	22.5±2.6	21.1±2.2
TC, mmol/L	4.26±0.74	4.19±0.79	4.31±0.85
TG, mmol/L	1.25±0.37	1.41±0.39	1.38±0.47
HDL-C, mmol/L	1.13±0.27	1.09±0.23	1.26±0.42
LDL-C, mmol/L	2.45±0.34	2.46±0.43	2.35±0.42

Values are mean ± SD. BMI, Body mass index; TC, Total cholesterol; TG, Triglycerides; HDL-C, High-density lipoprotein cholesterol; LDL-C, Low density lipoprotein cholesterol.

### *ABCG1* promoter SNP rs57137919G>A correlated with varying gene expression in PBMC-derived macrophages

It is possible that a promoter SNP can alter gene expression [18]–[20]. The effect of ABCG1 promoter SNP -376C>T on gene expression has been reported by Schou et al. [20]. The overall aim of the work was to provide functional evidence for the correlation of rs57137919 with atherosclerosis and ascertain whether the ABCG1 promoter SNP would result in any phenotypic polymorphisms caused by the alteration in molecular levels. Therefore, we first examined the effects of rs57137919 on ABCG1 and related gene expression in macrophages from healthy subjects carrying three genotypes.

**Figure 2:**
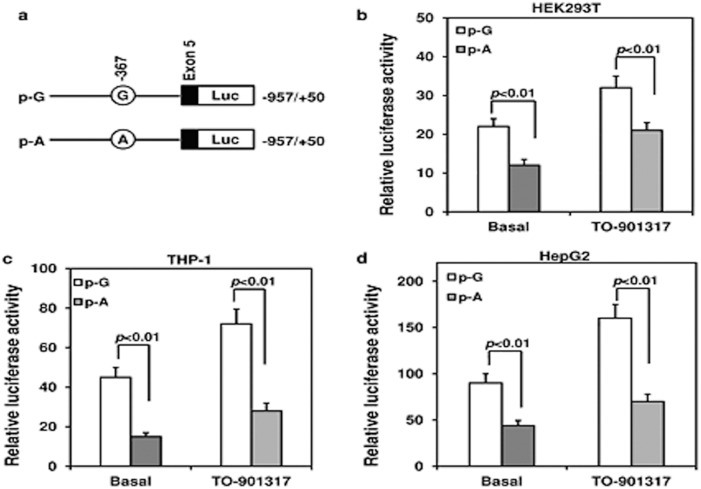
*ABCG1*/*ABCA1*/*SR-B1* gene expression in macrophage with G/G, G/A, and A/A genotypes of rs57137919. *ABCG1* mRNA (a) and *ABCA1* mRNA (b) levels in macrophages with G/G, G/A, and A/A genotypes of rs57137919. GAPDH was used as an internal control. Data are expressed as mean ± SD. Each experiment was performed in triplicate. *ABCG1* (c), *ABCA1*, and *SR-B1* (e) protein expression were detected by Western blotting. Representative pictures of protein expression are shown and densitometric data of bands of ABCG1 (d), ABCA1 (f), and SR-B1 (g) proteins normalized by β-actin. Data are expressed as mean ± SD from 8–10 independent experiments with monocytes from 8–10 different donors. n.s. indicates no statistical significance.

ABCG1 mRNA expression was significantly lower in macrophages from subjects with the A/A (0.41±0.10, n = 9, *P*<0.01) and G/A genotypes (0.87±0.04, n = 22, *P*<0.01) than from subjects with the G/G genotype (n = 25). ABCA1 mRNA expression exhibited no significant difference among the three subject groups (G/A: 1.07±0.07, n = 22; A/A: 1.15±0.08, n = 9; G/G: n = 25) (Figure 2a, b).

Western blot analysis showed ABCG1 protein level significantly lower in macrophages from A/A subjects (n = 9) compared with that from G/G subjects (n = 10, *P*<0.01). G/A subjects (n = 8) also showed a significantly lower ABCG1 protein level as compared to G/G subjects (*P*<0.05). While ABCA1 and SR-B1 protein levels showed no difference among the G/G, G/A, and A/A groups (G/G, n = 10; G/A, n = 8; A/A, n = 9) (Figure 2c–g). These results demonstrated rs57137919-dependent differences in *ABCG1* gene expression, suggesting that rs57137919A-carrying cells had significantly lower ABCG1 mRNA and protein expression levels.

### *ABCG1* rs57137919G>A inhibits promoter activity

To further investigate the role of rs57137919 in the regulation of *ABCG1* gene expression, we measured the luciferase reporter activity of the ABCG1 promoter construct containing the substitution in different cell lines and conditions.

In HEK293T cells, the luciferase reporter gene activity driven by the rs57137919A-containing promoter sequence construct (p-A) was significantly lower than that driven by the rs57137919G construct (p-G) (12±1.5 vs. 22±2, n = 4, *P*<0.01). Luciferase reporter gene activity driven by both p-A and p-G increased after TO901317 (2 µM) stimulation, although p-A activity was still significantly lower than that of p-G (21±2 vs. 32±3, n = 4, *P*<0.01) (Figure 1b). In THP-1 cells, reporter gene activity of p-A was significantly lower compared with p-G (15±2 vs. 45±5, n = 4, *P*<0.01); this was also the case after TO901317 (2 µM) stimulation (28±4 vs. 72±7.5, n = 4, *P*<0.01) (Figure.2c). In HepG2 cells, relative luciferase activity of the p-A construct was significantly lower than that of the wild-type p-G construct (44±5.5 vs. 90±10, n = 4, *P*<0.01), and the difference was greater after TO901317 intervention (70±8 vs. 160±15, n = 4, *P*<0.01) (Figure 1d). The results demonstrated that rs57137919A can inhibit ABCG1 promoter activity under both basal state and LXR agonist stimulation, which was consistent with the allele-dependent expression in macrophages (Figure 2).

### *ABCG1-*367G>A polymorphism impairs transcription factor binding activity

It has been reported that gene expression is regulated by affecting the binding of modulatory factors to the promoter region [18]. To investigate the molecular mechanism by which rs57137919A inhibits ABCG1 promoter activity and expression, we performed EMSA to evaluate the effect of ABCG1 rs57137919G>A polymorphism on nuclear protein binding. The results showed that *in vitro* nuclear protein (monocyte-derived macrophages and THP-1 cell nuclear extracts) can specifically bind to the promoter region around rs57137919.

**Figure 3:**
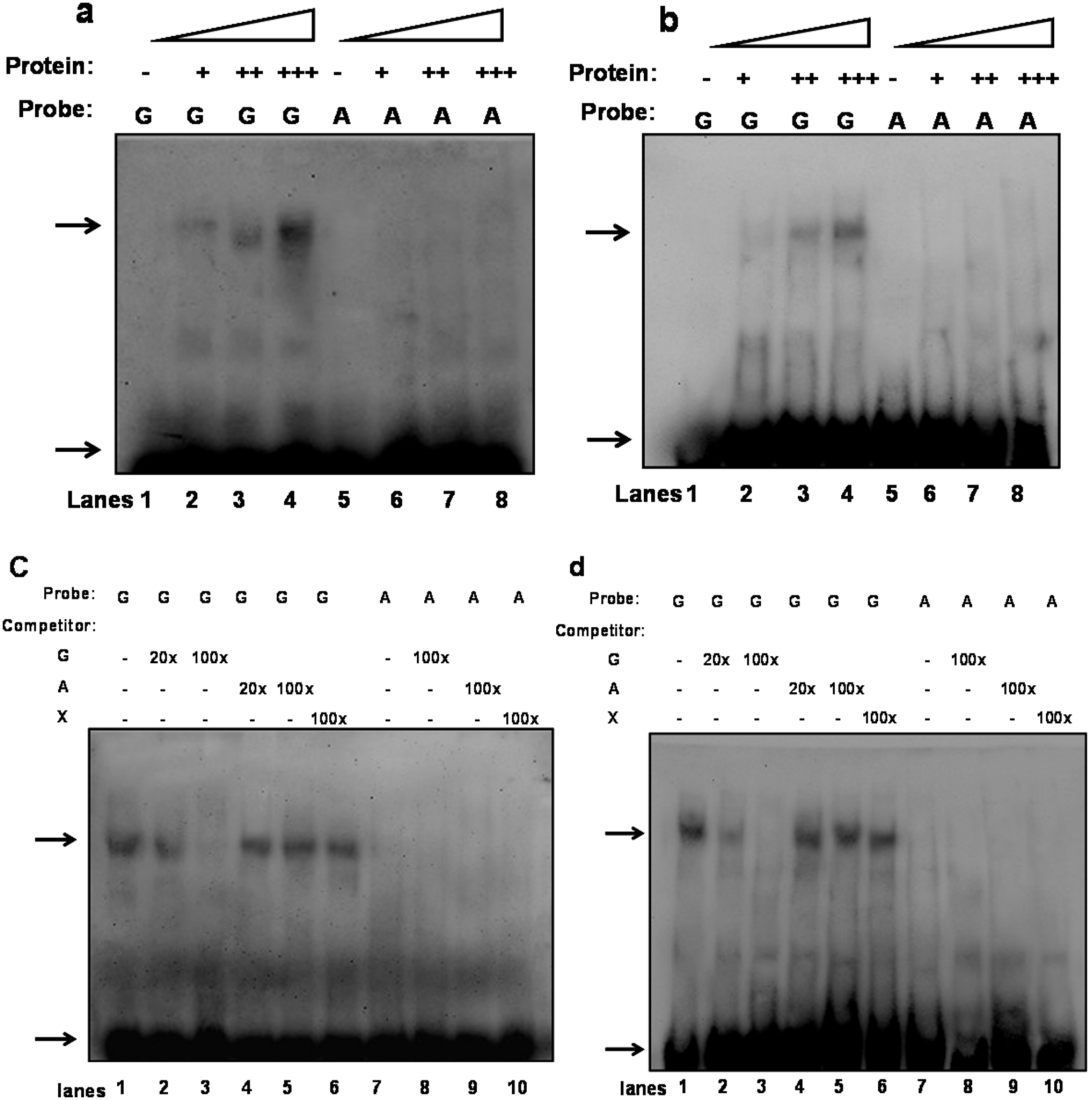
EMSA was performed to quantitatively analyse DNA-protein interaction. All samples contained binding buffer (1.5 µL), Poly dI:dC (1.5 µL), G allele or A allele probe (0.3 pmol), and nuclear protein extracts (5–15 µg). Band designations: upper arrow, protein-DNA probe complex; lower arrow, free DNA monocyte-derived macrophages (a) or THP-1 cell (b) nuclear protein bound to labelled DNA probe, and lanes 1–4 and lanes 5–8 contained 0, 5, 9, and 15 µg protein, respectively. Unlabelled cold probe competitively bound monocyte-derived macrophages (c) and THP-1 cell (d) nuclear protein. Specificity of binding was tested by competition with a 20- or 100-fold molar excess of unlabelled DNA probe added to the reaction mixture. Lanes 1 and 7: No competitor; lanes 2, 3, 9: self-competitor; lanes 4–6, 8, 10: cross-competitor. The results are representative of several independent experiments. Probe “G” for rs57137919G and “A” for rs57137919A biotin-labelled probes. Competitor “G” for rs57137919G, “A” for rs57137919A, and “X” for non-specific unlabelled probes.

Using the probe corresponding to the G allele, one major band of DNA-protein complex was detected. Its band intensity was gradually enhanced in proportion to the increased nuclear protein concentration (Figure 3a, b) and markedly reduced in the presence of the unlabelled G allele as shown in the competitive binding assay with an unlabelled self- or cross-competitive DNA probe (Figure 3c–d). There was no major band detected in the assay using the probe sequences containing the rs53197919A allele. These results indicated that *ABCG1-*367G>A variant significantly inhibited the binding between nuclear protein and DNA.

These data, together with the results of ABCG1 expression, suggested that the promoter sequence containing rs57137919A allele can inhibit *ABCG1* gene expression, at least partly, by altering DNA-protein interactions in the promoter region.

### Decreased *ABCG1*-mediated cholesterol efflux from macrophages to HDL

**Figure 4:**
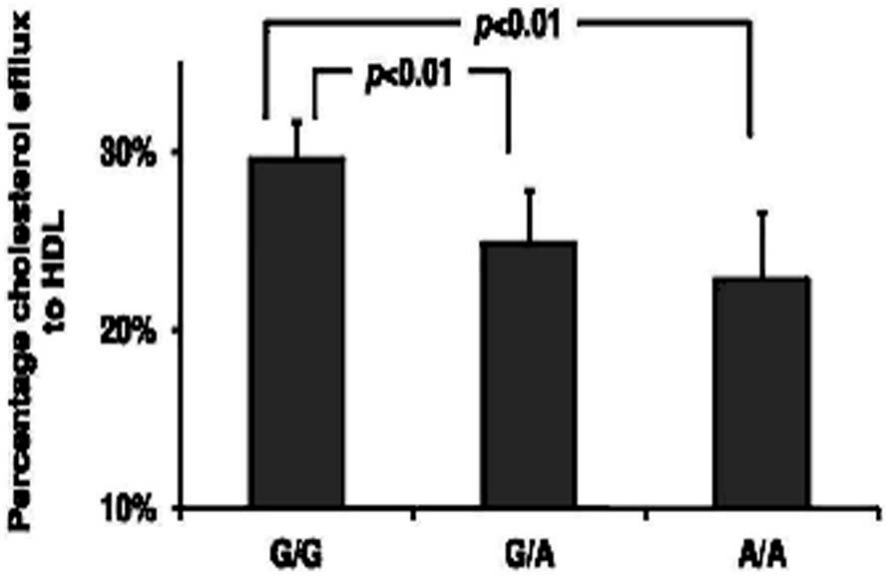
ABCG1-mediated cholesterol efflux from monocyte-derived macrophages to HDL. PBMCs were isolated from subjects and differentiated into macrophages using 20/mL M-CSF for 7 days. ABCG1-mediated cholesterol efflux was induced by 50 µg/mL HDL and cholesterol efflux rates were measured using NBD fluorescence assay. Values are expressed as means ± SD (n = 9–15 donors/genotype). Each sample was repeated three times and averaged.

To evaluate the effect of *ABCG1* rs57137919G>A polymorphism on gene function, we measured net cholesterol efflux to HDL from macrophages. The ABCG1-mediated percentage cholesterol efflux of PBMC-derived macrophages from A/A subjects (22.9±3.8%, n = 9, *P*<0.01) or G/A subjects (24.9±2.9%, n = 11, *P*<0.01) was lower than that from G/G subjects (29.6±2.1%, n = 15). There was no significant difference with respect to the cholesterol efflux between A/A and G/A subjects (*P*>0.05) (Figure 4). These results indicate that the *ABCG1* rs57137919G>A polymorphism decreased ABCG1-mediated cholesterol efflux from human macrophages probably by downregulating ABCG1 expression.

### Increased apoptosis and proapoptotic gene levels of ox-LDL loaded macrophages in subjects with *ABCG1* rs57137919A allele

Apoptosis plays an important role in atherosclerotic development. In order to explore the possible mechanism by which the rs57137919G>A polymorphism affects atherosclerotic development, we hypothesized that the decreased atherosclerosis in human may result from the increase in macrophage apoptosis in response to ox-LDL.

**Figure 5:**
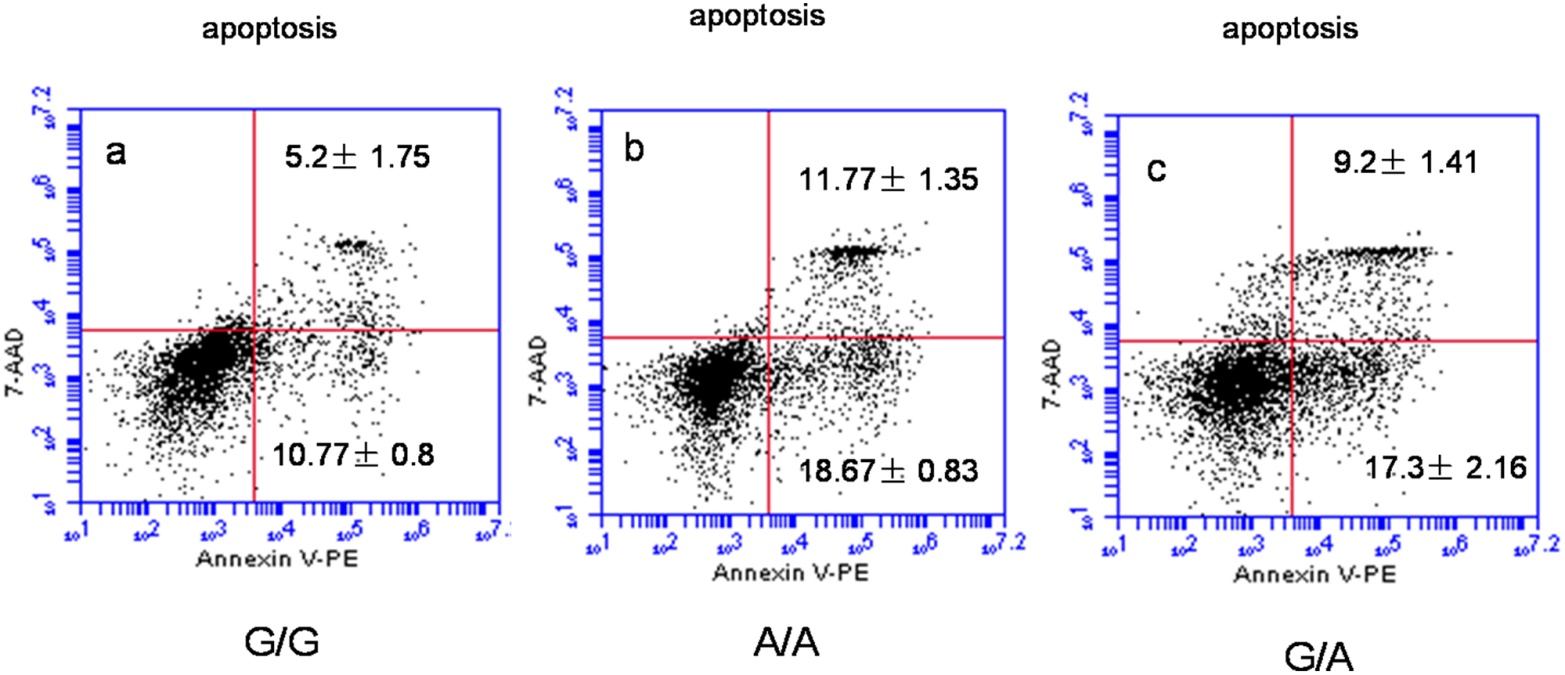
*ABCG1* rs57137919G>A polymorphism promoted macrophage apoptosis. After loading of 50 µg/mL ox-LDL for 24 hours, the apoptosis of PBMC-derived macrophages was measured by flow cytometry. Annexin V-PE/7-AAD double-staining assay was used to quantify apoptosis in human macrophages by flow cytometry. Macrophage apoptosis includes early apoptotic (PE+7AAD−) and late apoptotic (PE+7AAD+). (a) Apoptosis in G/G genotype macrophages. (b, c) Increased apoptosis in A/A genotype and G/A genotype macrophages. Values are mean ± SD. Representative images are shown for nine independent experiments with monocytes from different donors.

To test this hypothesis, we performed flow cytometry-based apoptosis analysis to determine the association between the rs57137919G>A polymorphism and the apoptosis of human PBMC-derived macrophages. After loading 50 µg/mL ox-LDL for 24 hours, the apoptosis of PBMC-derived macrophages was measured by flow cytometry. Apoptosis was significantly accelerated in macrophages with the A/A (30.44±2.18%, n = 9, *P*<0.01) and G/A genotypes (26.5±3.57%, n = 9, *P*<0.01) of the rs57137919G>A polymorphism compared to those with the G/G genotype (15.97±2.55%, n = 9). There was no significant difference between the level of apoptosis in macrophages from A/A and G/A subjects (*P*>0.05) (Figure 5).

**Figure 6:**
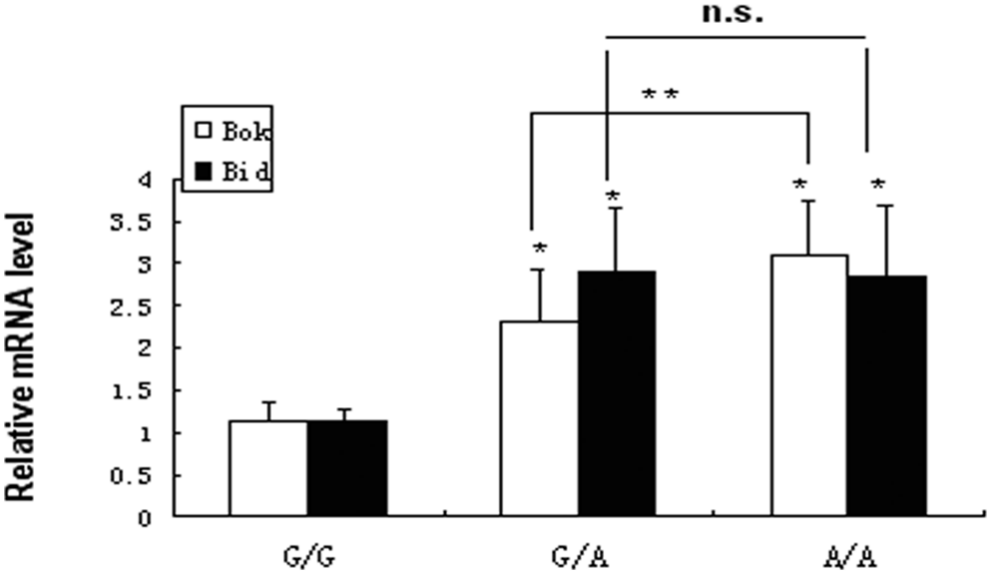
Apoptosis-associated genes Bok and Bid mRNA expression in monocyte-derived macrophages. PBMCs-derived macrophages from three genotypes were exposed to 50 µg/mL ox-LDL for 24 hours and RNA was extracted using TRIzol regent. Bid and Bok mRNA levels were detected by quantitative real-time PCR. Data are expressed as mean ± SD from nine donor samples in triplicate. *P<0.001 vs. G/G control, **P<0.05 between G/A and A/A; n.s. indicates no statistical significance.

The increased sensitivity of macrophages with rs57137919A allele to oxLDL-induced apoptosis suggested that these macrophages might also exhibit variant expression of apoptosis-related genes. Therefore, we also performed quantitative RT-PCR to identify apoptotic genes in PBMC-derived macrophages after exposure to ox-LDL. The results showed that *Bid* mRNA level was significantly increased in macrophages from A/A (2.85±0.83, n = 9, *P*<0.01) and G/A subjects (2.9±0.75, n = 9, *P*<0.01) compared with that from G/G subjects (1.14±0.15, n = 9). The *Bok* mRNA level was also increased in A/A (3.09±0.65, n = 9, *P*<0.01) and G/A macrophages (2.31±0.62, n = 9, *P*<0.01) as compared to that in G/G macrophages (1.14±0.21, n = 9) (Figure 6). The evidence of rs57137919G>A polymorphism on macrophage apoptosis was further strengthened by analysing the mRNA level of *Bid* and *Bok*, two members of the *Bcl-2* proapoptotic gene family, which were shown to have increased expression in A/A and G/A genotype macrophages.

Based on the apoptosis study data, we observed that rs57137919A-carrying macrophages were more susceptible to apoptosis after exposure to ox-LDL than rs57137919G-carrying macrophages. These data strongly indicate rs57137919G-dependent attenuation and rs57137919A-dependent enhancement of apoptosis of macrophages in the presence of ox-LDL. Therefore, we conclude that rs57137919A-associated apoptosis may provide functional evidence for the reduced risk for CAD in subjects with *ABCG1* promoter rs57137919A allele, as previously reported [14].

## Discussion

Cholesterol efflux from macrophages is one of the most critical steps in the process of reverse cholesterol transport and plays an important role in anti-atherosclerosis. ABCG1 is a member of the ABC superfamily of transmembrane transporters. Both *in vitro* and *in vivo* studies have shown that ABCG1 mediates cellular cholesterol efflux by transporting cholesterol to mature HDL [21], [22]. Although some studies have demonstrated the anti-atherosclerotic effect of ABCA1, the role of ABCG1 in atherosclerosis remains controversial, mostly because of inconsistent results from animal studies [10]–[12], [23], [24].

Our previous study showed that the SNP rs57137919G>A in the ABCG1 promoter region was associated with reduced risk for atherosclerotic CAD and ABCG1 expression, suggesting that ABCG1 expression might be atherogenic in human [14]. Herein, we try to provide functional evidence to understand the role of the rs57137919G>A polymorphism in atherosclerosis in human.

In this study, we increased the sample size to further demonstrate that the ABCG1 promoter variant, rs57137919G>A, could lead to a notable difference in gene expression. In order to more comprehensively verify the effect of rs57137919G>A on the regulation of ABCG1 promoter activity, we measured luciferase reporter gene expression driven by the ABCG1 promoter with rs57137919G or A alleles under a basal state as well as under LXR agonist (TO901317) stimulation in HEK293, HepG2, and THP-1 cells. The results clearly showed that the inhibition effect of rs57137919A on ABCG1 promoter activity was more potent under LXR agonist stimulation in different cell lines, suggesting that the activation of the *ABCG1* gene promoter driven by LXR agonist might be suppressed by the rs57137919A allele. It is widely accepted that oxysterols, as the ligands for LXR activation, can activate the *ABCG1* promoter and upregulate ABCG1 transcription in LXR-dependent mechanisms [25]. Based on our findings, it is reasonable to speculate that the rs57137919A allele can also exert its inhibition effect on LXR-dependent ABCG1 expression activated by the ox-LDL loading of macrophages.

In our previous study using the PATCHTM public 1.0 prediction software, we predicted that putative regulatory elements, VDR and GAGA factor, may bind to site rs57137919 in the ABCG1 promoter region [14]. In the present study, EMSA clearly showed that the wild-type G site probes, rather than mutant A site probes, were capable of specifically binding the protein extracted from PBMC-derived macrophages or THP-1 cell nuclei, suggesting that the rs57137919G>A variant would weaken the binding between the ABCG1 promoter and the transcription factor, and consequently reduce ABCG1 promoter transcription. Based on these results, it is plausible that the differences in *ABCG1* gene expression may have resulted, at least partly, from the modulatory effect of the various rs57137919 sequences on DNA-protein interactions in the promoter region. However, whether the phenotypic outcomes are relevant to DNA-protein interactions is yet to be verified. Therefore, it will be beneficial to conduct additional studies to substantiate the regulatory factors and mechanisms that might be involved.

Previous *in vitro* studies have suggested that ABCG1 is responsible for sterol efflux from cholesterol-loaded macrophage foam cells to mature HDL [6], [7], [26], [27]. Further, Wang et al. reported that macrophages lacking ABCG1 expression impaired cholesterol efflux to HDL and significantly reduced reverse cholesterol transport *in vivo* [7]. Studies in cell lines showed that ABCG1, rather than ABCA1, can specifically mediate 7-ketocholesterol [28] and 7β-hydroxycholesterol [28], [29] efflux from cells to HDL. These are two oxysterols existed in oxidized LDL with the oxidation at C7-position. Within human atherosclerotic lesions, 7-ketocholesterol and 7β-hydroxycholesterol exerted cytotoxic effects in promoting macrophage apoptosis [30], [31]; similar findings were reported in studies using *ABCG1* ^−/−^ mice [28], [32]. Herein, we found that the ABCG1 promoter rs57137919A was associated with a significantly downregulated ABCG1 expression and attenuated cholesterol efflux, which may have led to the accumulation of specific oxysterols in macrophages and accelerated cell apoptosis.

Macrophage apoptosis plays a critical role in the development of atherosclerosis [33]–[35]. In fatty streak lesions, which form the early stage of atherosclerosis, an increase in macrophage apoptosis is atheroprotective [34], [35], while in advanced atherosclerotic lesions, an increase in macrophage apoptosis leads to necrotic core development, contributing to vulnerable plaque formation and thrombosis [35]. This study demonstrated that the ABCG1 promoter rs57137919G>A was associated with increased macrophage apoptosis, which may probably be caused by the accumulation of oxysterol in macrophages due to the attenuated ABCG1-mediated cholesterol efflux. Baldan et al. observed an impaired development of atherosclerosis in *Ldlr^−/−^* mice transplanted with *Abcg1*−/− bone marrow, in which atherosclerotic lesions contained increased numbers of apoptotic cells [11]. *In vitro*, Baldan et al. found that after exposure to ox-LDL, *Abcg1* ^−/−^ macrophages were more susceptible to apoptosis than *Abcg1* ^+/+^ cells [11]. Similarly, Tarling et al. reported that *Abcg1* ^−/−^ *Apoe* ^−/−^ mice exhibited smaller atherosclerotic lesions and more prominent macrophage apoptosis [32]. Together with the findings of the present study, we considered that the proapoptotic effect of the ABCG1 promoter rs57137919G>A polymorphism was probably due to the accumulation of cytotoxic oxysterols in macrophages. These results provide a possible explanation for the potentially atherogenic role of ABCG1 expression in humans.

In addition to macrophages, ABCG1 is also highly expressed in endothelial cells, which play a role in cholesterol homeostasis to avoid endothelial activation in the vessel wall [36]. Previous studies demonstrated that *Abcg1* ^−/−^ mice fed a Western diet accumulated the oxysterol in endothelial cells and reduced active eNOS dimer levels [37]. A study by siRNA transfection in human umbilical artery endothelial cells showed that ABCG1 deficiency promoted endothelial cell apoptosis *in vitro* by an endoplasmic reticulum stress-dependent pathway [38]. In contrast, Tarling et al. proposed that endothelial cells from *Abcg1* ^−/−^ mice did not show accelerated apoptosis *in vivo* [32]. Due to the lack of observation in our study, whether the anti-atherosclerosis role of rs57139719G>A mutation is functionally related to endothelial cells remains unknown. In addition, whether the rs57137919G>A variant-associated lower risk of CAD found in our previous study is simply an outcome of the increased apoptotic macrophages in lesions or of the smaller lesions remains to be investigated and an animal model for the role of the promoter variant should be considered.

In conclusion, through the use of human PBMC-derived macrophages as well as various cell lines, this study has shown that a promoter SNP in ABCG1 is functionally interrelated with the gene expression and apoptosis of macrophages, leading to a phenotypic difference and a cause for atherosclerosis. Therefore, this study furthers the understanding of the role of ABCG1 in atherosclerosis and its potential value as a disease marker.
