# Correction: ABCG1 rs57137919G>A Polymorphism Is Functionally Associated with Varying Gene Expression and Apoptosis of Macrophages

**DOI:** 10.1371/journal.pone.0108083

**Published:** 2014-09-05

**Authors:** The PLOS ONE Staff

The first and second authors, Fang Liu and Wei Wang, should be noted as contributing equally to this work.

In the Author Contributions section, Xiao-Wei Yan (XWY) should be listed as one of the persons who analyzed the data.
